# Electro-haptic stimulation enhances speech recognition in spatially separated noise for cochlear implant users

**DOI:** 10.1038/s41598-020-69697-2

**Published:** 2020-07-29

**Authors:** Mark D. Fletcher, Haoheng Song, Samuel W. Perry

**Affiliations:** 1 University of Southampton Auditory Implant Service, University of Southampton, University Road, Southampton, SO17 1BJ UK; 2 Faculty of Engineering and Physical Sciences, University of Southampton, University Road, Southampton, SO17 1BJ UK

**Keywords:** Auditory system, Human behaviour, Perception, Translational research

## Abstract

Hundreds of thousands of profoundly hearing-impaired people perceive sounds through electrical stimulation of the auditory nerve using a cochlear implant (CI). However, CI users are often poor at understanding speech in noisy environments and separating sounds that come from different locations. We provided missing speech and spatial hearing cues through haptic stimulation to augment the electrical CI signal. After just 30 min of training, we found this “electro-haptic” stimulation substantially improved speech recognition in multi-talker noise when the speech and noise came from different locations. Our haptic stimulus was delivered to the wrists at an intensity that can be produced by a compact, low-cost, wearable device. These findings represent a significant step towards the production of a non-invasive neuroprosthetic that can improve CI users’ ability to understand speech in realistic noisy environments.

## Introduction

Cochlear implants (CIs) are neuroprosthetic devices that enable profoundly hearing-impaired people to perceive sounds through electrical stimulation of the auditory nerve. They have been remarkably successful, allowing many users to achieve excellent speech recognition in quiet environments^1^. However, CIs still have substantial limitations, particularly in environments such as classrooms, cafes, and busy workplaces, where users struggle to separate speech from background noise^2,3^ and to discriminate sounds coming from different locations^4^. Traditionally, researchers have attempted to overcome these limitations by improving CI technology and surgical techniques. However, in recent decades, these attempts have not led to substantial improvements in patient outcomes^1,5^. In the current study we take a new approach, using a second, non-invasive haptic neuroprosthetic to augment the CI signal. We use haptic stimulation to complement the electrical CI signal by providing missing or degraded speech and location information. We tested whether this “electro-haptic stimulation” (EHS)^6–10^ can enhance speech-in-noise performance for CI users when speech and noise come from different locations.

In normal-hearing listeners, having access to spatial hearing cues makes speech much easier to understand when a masking sound is coming from a different location^11–13^. The difference in arrival time and sound intensity between the ears (interaural time and level differences) are crucial cues for determining the location of a sound. CI users have limited access to these cues, especially the around 95%^14^ of users who are implanted in only one ear^4,15^. As a result, CI users have smaller improvements in speech-in-noise performance than normal-hearing listeners when the speech and noise are spatially separated, particularly those that have only one implant^16–21^. Recently, Fletcher et al.^8^ showed that EHS can enhance sound localization in CI users. After just 15 min of training, EHS was found to allow CI users to locate sounds with similar accuracy to hearing-aid users. This improved ability to locate a single sound suggests that CI users may be able to more effectively separate multiple sounds coming from different locations, resulting in improved speech-in-noise performance.

Some previous studies have shown evidence that speech-in-noise performance can be enhanced in CI users by presenting speech information through haptic stimulation for sounds from the same location^6,7,9^. However, in most real-world scenarios, target and masking sounds do not come from the same location. In the current study, we presented speech amplitude envelope cues, extracted from the signals that would be received by behind-the-ear hearing aids or CIs, and delivered them to each wrist through haptic stimulation. This meant that the intensity differences between the wrists corresponded to the intensity differences between the ears. The amplitude envelope of the speech was extracted at frequencies where interaural level difference cues are large and there is significant speech energy. These envelopes were then remapped to a frequency range where the skin is most sensitive to haptic stimulation (see Methods). By presenting spatial and speech-envelope cues that may be used for auditory object identification and separation, and by allowing the user to always have access to a haptic signal from the ear with the best signal-to-noise ratio (SNR), we aimed to improve speech-in-noise performance for spatially separated sounds in CI users. Our approach was designed to be readily transferrable to a real-world application, with the haptic signal processing performed in real-time and the signal presented at an intensity that could readily be produced by a compact, wearable neuroprosthetic device.

Speech-in-noise performance was measured with and without concurrent haptic stimulation in nine CI users, each of whom was implanted in only one ear. Measurements were made when the speech and multi-talker noise were in the same location (both directly in front) or in different locations, with speech directly in front and the noise either presented from the side with the implant (ipsilateral) or from the opposite side (contralateral). Before testing, participants were trained for around 30 min with and without haptic stimulation. We expected little or no enhancement with EHS when the noise was in the same location as the speech. This condition was similar to a previous study that found an EHS enhancement effect using noise reduction^6^. However, in the current study, no noise reduction was applied to avoid distortion of spatial hearing cues. In contrast, when speech and noise were spatially separated (with the noise either ipsilateral or contralateral to the CI), an EHS enhancement effect was expected. In these conditions, participants always had access to speech envelope cues through haptic stimulation on the wrist corresponding to the ear opposite the noise position. On this wrist, the SNR would be improved by the reduction in noise energy caused by the acoustic shadow of the head. Because of the frequency dependence of the head shadow^30^, this reduction in noise energy would be expected to be largest at higher frequencies, where the haptic signal-processing was focused. Furthermore, there is evidence that knowledge of target speech or tone location can improve detection and recognition of spatially separated sounds, by allowing listeners to optimally focus attention^22–24^. It is possible that providing information about the location of the speech and noise stimuli (which is severely degraded for CI users) through haptic stimulation, will also improve performance by optimizing the focus of attention.

## Results

**Figure 1:**
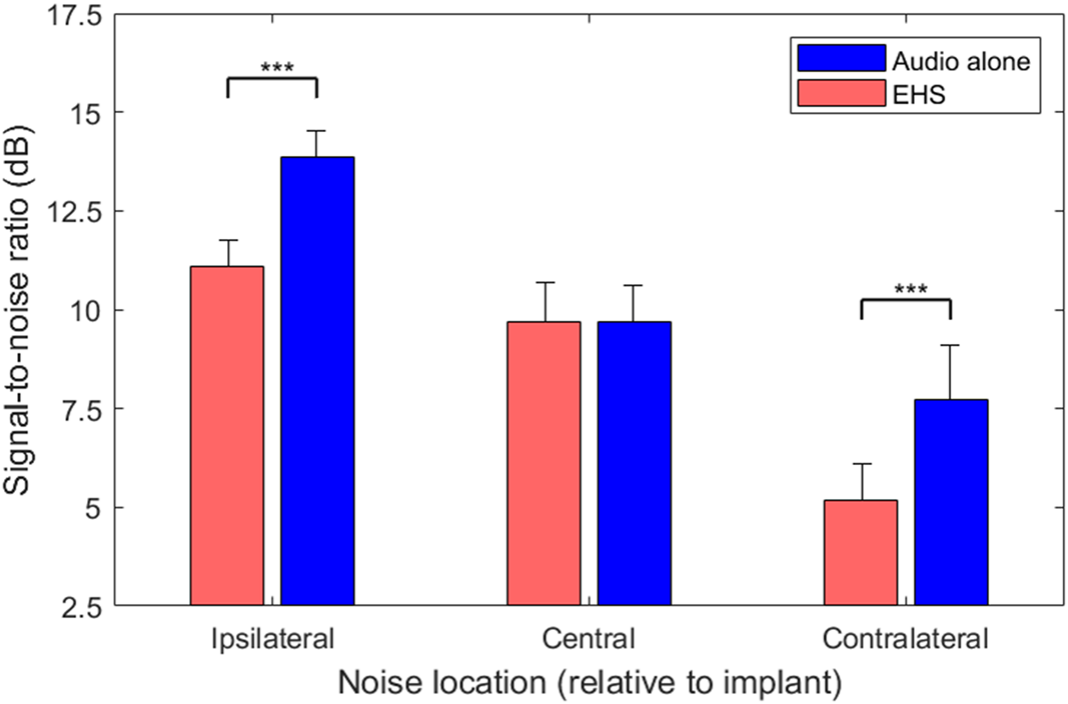
Speech-in-noise performance with and without EHS for each noise position (central, ipsilateral or contralateral to the implant). Lower signal-to-noise ratios indicate better performance. Error bars show the standard error of the mean.

Figure 1 shows speech-in-noise performance with and without EHS for a multi-talker noise at the same location as the speech (centre) or at a different location (either ipsilateral or contralateral to the participant’s implant). Significant overall effects of EHS (*F*(1.00,8.00) = 66.7, *p* ≤ 0.001) and noise location (*F*(1.47,11.77) = 33.7, *p* ≤ 0.001) were found. A significant interaction between the effect of EHS and the location of the noise was also found (*F*(1.23,9.81) = 12.3, *p* = 0.001), indicating that the effect of EHS was different for different noise locations.

Three planned post-hoc *t*-tests were conducted (with a Holm-Bonferroni correction for multiple comparisons). EHS was found to improve performance when the speech and noise were spatially separated. When the noise was ipsilateral to the participant’s CI, EHS was found to improve the speech reception threshold (SRT) in multi-talker noise by 2.78 dB on average (*t*(8) = 6.6, *p* ≤ 0.001), reducing the SRT from 13.85 to 11.08 dB. EHS was also found to improve the SRT in noise when the noise was contralateral to the participant’s CI (*t*(8) = 4.6, *p* ≤ 0.001); the SRT was reduced by 2.55 dB on average, from 7.74 to 5.19 dB. No effect of EHS was found when the noise was in presented from the same central location as the speech (*t*(8) = 0.09, *p* = 0.930; mean difference: 0.02 dB). Additional post-hoc analyses were also performed (corrected for multiple comparisons) to establish whether there was a correlation between the SNR for the audio-only condition and the amount of EHS benefit for each noise location.

**Figure 2:**
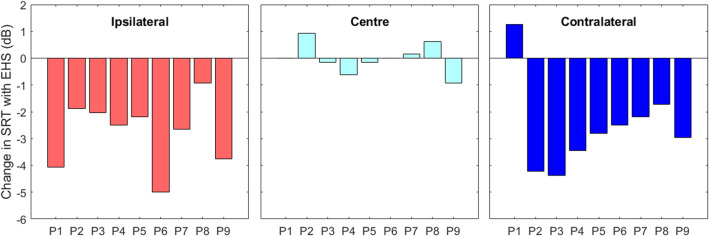
Change in speech reception threshold (SRT) in multi-talker noise with EHS, for each participant. A negative change indicates that EHS improved performance compared to audio alone. Panels show effects when the noise was either in the same central location as the speech (central panel, light blue bars) or was ipsilateral (left panel, red bars) or contralateral (right panel, dark blue bars) to the participant’s CI.

Figure 2 shows the improvements in performance with EHS for each noise position in each participant. For the ipsilateral noise position, EHS improved performance in all participants, with improvement ranging from 0.94 to 5 dB. For the contralateral noise position, EHS improved performance in all but one participant, with effects ranging from – 1.25 to 4.38 dB. Six out of the nine participants showed an EHS benefit of 3 dB or more for either the ipsilateral or contralateral noise. For the central noise position, the change in SRT with EHS ranged from – 0.94 to 0.94 dB.

## Discussion

In this study, we found that EHS gave a robust improvement in speech recognition in multi-talker noise for spatially separated sounds. These findings were made in CI users with one implant, who make up around 95% of the CI community. This improvement was present both when the multi-talker noise was ipsilateral to the implant, where the mean improvement in the SRT was 2.8 dB, and when the noise was contralateral to the implant, where the mean SRT improvement was 2.6 dB. These improvements were observed across users of three different cochlear implant systems, after just half an hour of training with EHS. These findings confirm the hypothesis that CI users can more effectively separate multiple sounds coming from different locations when speech and spatial cues are provided through haptic stimulation.

Two previous studies have measured EHS benefit to speech-in-noise performance for co-located speech and noise. The current study found slightly larger benefit to speech-in-noise performance than previous studies that have used EHS^6,7^. In contrast to the current study, that presented speech envelope cues, Haung et al.^7^ presented the fundamental frequency of speech through haptic stimulation. They found an average improvement in SRT of 2.2 dB in noise, which is smaller than the improvements measured in the current study. That the improvement found was larger than in Haung et al. is encouraging, as the current approach is more readily adaptable to a real-world application. In particular, in the current study, haptic stimulation was delivered to the wrist rather than the fingertip and the haptic signal was extracted from speech-in-noise in real-time, rather than from the clean speech with offline processing.

Two sub-groups of CI users have been shown to have enhanced speech-in-noise performance for spatially separated sounds. One group are those that are able to retain residual low-frequency acoustic hearing after implantation^25–27^. This group shows a similar benefit to that shown for EHS in the current study. Unfortunately, few patients referred for CI fitting have usable residual hearing^28^, so most are unable to access these benefits. A second sub-group are those who have a second implant fitted. Two studies have shown that having two implants active rather than one improves speech-in-noise performance. In both studies, speech was presented in front of the listener; the noise was presented either ipsilateral or contralateral to the implant used in the condition where only one implant was active^18,19^. Having a second active implant was found to improve the SRT in noise by ~ 5 dB for an ipsilateral noise and ~ 2 dB for a contralateral noise. This compares to our findings of 2.8 dB and 2.6 dB improvements respectively for EHS. It should be noted, however, that in both studies that examined the benefits of a second implant, all participants used two implants in their daily lives. This meant that participants were highly trained using both of their CIs, but not using a single CI. This may have led to exaggerated estimates of the benefit of using two CIs. Conversely, in the current study, participants listened without EHS in their daily lives and received only around 30 min of EHS training. This may have led to an underestimate of the potential benefits of EHS.

When the speech and noise were presented from the same location, no effect of EHS was found. This demonstrates that the benefits of EHS are not due to a placebo effect, caused either by the novelty of haptic stimulation or the expectation that EHS will improve performance. The condition with co-located speech and noise is similar to the condition tested in Fletcher et al.^6^ who, unlike in the current study, found a benefit of EHS. In Fletcher et al., the speech amplitude envelope was extracted from audio between 100 and 1,000 Hz, where most energy in speech is focused^29^. In the current study, however, the envelope was extracted between 1,000 and 10,000 Hz, where interaurual level difference cues are large^30^ and there is still substantial speech energy. It seems unlikely that this difference is critical to allowing participants to benefit from speech envelope cues as the current study found clear EHS benefit when the speech and noise were spatially separated. A potentially important difference between the current study and Fletcher et al. is the use of an expander in Fletcher et al.’s signal-processing chain. This was used for noise reduction and to exaggerate the speech amplitude envelope modulations. Given that the use of an expander appears to be a key difference between the two signal-processing approaches, it seems likely that the expander was important for producing EHS benefit for co-located sounds. Future work should seek to combine the current approach with a noise-reduction technique that does not distort spatial hearing cues to maximize benefit for both co-located and spatially separated signals.

There are a number of mechanisms through which EHS may have improved speech-in-noise performance. One possibility is that a better SNR was available to the haptic device than to the CI. In the ipsilateral noise condition, a better SNR was available to the haptic device on the side contralateral to the CI. However, a better SNR was not available in the central noise condition, where the haptic-device SNR on either side was equal to that of the CI. It was also not available in the contralateral noise condition, where the side with the best SNR was the same for the CI and haptic device. Therefore, EHS benefit would be expected for the ipsilateral condition, but not for the central or contralateral conditions. As expected, EHS benefit was measured for the ipsilateral condition but not the central condition. However, for the contralateral condition, an EHS benefit that matched that of the ipsilateral condition was found. One possible explanation for this discrepancy is that the haptic signal-processing may have led to an improved SNR for the haptic signal, despite the SNR available to the CI and haptic devices being the same. This could have occurred because, for the contralateral condition but not the central condition, the noise on the side with the best SNR was subject to acoustic head-shadowing. For the haptic device, the acoustic head shadow attenuated the noise used in the current study by 7 dB. The acoustic head-shadow is frequency dependent so that higher frequencies are attenuated more strongly than lower frequencies^30^. This could mean that the haptic signal, which was derived from higher audio frequencies than the CI signal, was less polluted by the noise. We assessed the difference in the amount of noise in the haptic signal on the best SNR side for all three noise conditions (using 100 sentence and noise samples from the current experiment for each condition). The haptic signal was found to have an average SNR of 18.6 dB for the ipsilateral condition, of 6.1 dB for the central condition, and of 12.5 dB for the contralateral condition. This worse SNR for the haptic signal in the central condition may explain why no EHS benefit was found. Further work is required to establish the relationship between the haptic-signal SNR and the amount of EHS benefit.

Another mechanism that may have contributed to improved speech-in-noise performance for the ipsilateral and contralateral noise conditions is attentional focusing. Interestingly, all participants reported that they believed EHS was beneficial because it indicated the locations of the speech and noise. Previous studies have shown evidence that knowledge of a target sound’s location can improve detection and recognition of spatially separated sounds by allowing the listener to optimally focus attention^22–24^. It is possible that a similar process occurred in the current study, with attention directed to the correct location by EHS. Future work should investigate the extent to which attentional focusing can account for the benefits of EHS to speech-in-noise performance for spatially separated sounds.

In the current study, visual information indicating the location of sounds and giving information through lip-reading cues was not provided. It has been shown that temporal properties of visual stimuli can bias the perceptual organization of an auditory scene^31^. Visual stimuli can also influence auditory object formation^32^ and how auditory cortical neurons respond to sound mixtures^33^ as well as the perception of sound location (as demonstrated in the ventriloquism effect)^34,35^. If more visual information were available in the current experiment, it may have made beneficial information about the auditory scene provided through haptic stimulation redundant (although note that the limited field of view restricts information about the location of sounds). Visual information could also have provided lip-reading cues, which improve speech-in-noise performance by giving information about the rhythmic and amplitude changes in speech^36^ and about the place of articulation^37^. Lip-reading cues can enhance the neural representation of the speech amplitude envelope both in quiet and in background noise^38–41^. It is therefore possible that lip-reading cues would provide much of the beneficial speech amplitude envelope information that was delivered through haptic stimulation. However, there is strong evidence that providing speech amplitude envelopes through haptic stimulation enhances lip reading^42,43^. The provision of both visual and haptic stimulation may therefore lead to greater benefits to speech-in-noise performance. Further work is required to establish the impact of visual information on EHS benefits.

This study has shown that EHS substantially improves speech-in-noise performance, when speech and noise come from different locations, after just 30 min of training. These improvements were shown for multi-talker background noise, in which CI users^1,44^ and noise-reduction algorithms^45,46^ struggle most. Our approach was devised so that it could easily be implemented in a system that could be used in the real world: the signal processing was computationally lightweight and was applied in real-time, the stimulation site was suitable for real-world application, and haptic stimulation was delivered at an intensity that could easily be achieved by a low-cost, low-power neuroprosthetic device. The findings of this study represent an important step towards providing a non-invasive and inexpensive means of substantially improving outcomes for CI users.

## Online methods

### Participants

Nine CI users (4 males and 5 females; a mean age of 50 years old, ranging from 23 to 67 years old) were recruited through the University of Southampton Auditory Implant Service. All participants were native British English speakers, had been implanted at least one year prior to the experiment, had speech-in-quiet scores of at least 70% (for Bamford–Kowal–Bench sentences; BKB), and had the capacity to give informed consent. Participants all had a CI in one ear and no device in the other ear. Participants had no residual acoustic hearing, defined here as having unaided thresholds at 250 and 500 Hz that are 65 dB HL or worse in either ear. Participants completed a screening questionnaire, confirming that they had no medical conditions and were taking no medication that may affect their sense of touch. Table 1 details the characteristics of the participants who took part in the study. Participants gave written informed consent and received an inconvenience allowance.

**Table 1:** Summary of participant characteristics.

Participant number	Sex	Age (years)	Device	Device side	Years since implantation
1	M	26	Cochlear CI512 (Contour Advance)	Left	1.8
2	F	60	MED-EL Synchrony Flex 28	Right	10.8
3	F	23	Advanced Bionics Hi Res 90 K	Right	10.7
4	F	67	Advanced Bionics Hi Res 90 K	Left	11.5
5	M	26	Cochlear CI512 (Contour Advance)	Left	1.3
6	F	59	Cochlear CI512 (Contour Advance)	Left	2.0
7	M	58	MED-EL Synchrony Flex 28	Right	3.7
8	M	63	Advanced Bionics Hi Res Ultra	Right	1.0
9	F	63	MED-EL Synchrony Flex 28	Right	10.2

Vibrotactile detection thresholds were measured at the fingertip and wrist at 31.5 Hz and 125 Hz following conditions and criteria specified in ISO 13091-1:2001^47^. All participants had vibrotactile detection thresholds within the normal range (< 0.4 ms^−2^ RMS at 31.5 Hz and < 0.7 ms^−2^ RMS at 125 Hz)^47^ based on fingertip measurements (mean at 31.5 kHz = 0.18 ms^−2^ RMS; mean at 125 Hz = 0.29 ms^−2^ RMS). The mean vibrotactile detection threshold on the wrist at 31.5 Hz was 0.93 ms^−2^ RMS, and at 125 Hz was 0.92 ms^−2^ RMS (averaged across left and right wrists; there are no published standards for normal wrist sensitivity).

### Stimuli

The speech stimuli were presented at a level of 65 dB SPL LAeq. Each of the three loudspeakers were calibrated at the listening position using a Brüel & Kjær (B&K) G4 type 2250 sound level meter (which was calibrated using a B&K type 4231 sound calibrator). The BKB speech corpus, with a male talker, was used for training and the Institute of Electrical and Electronic Engineers (IEEE) corpus, with a different male talker, was used for testing. A non-stationary multi-talker noise recorded by the National Acoustic Laboratories (NAL)^48^ was used in both training and testing sessions. The noise was recorded at a party and had a spectrum that matched the international long-term average speech spectrum.

**Figure 3:**
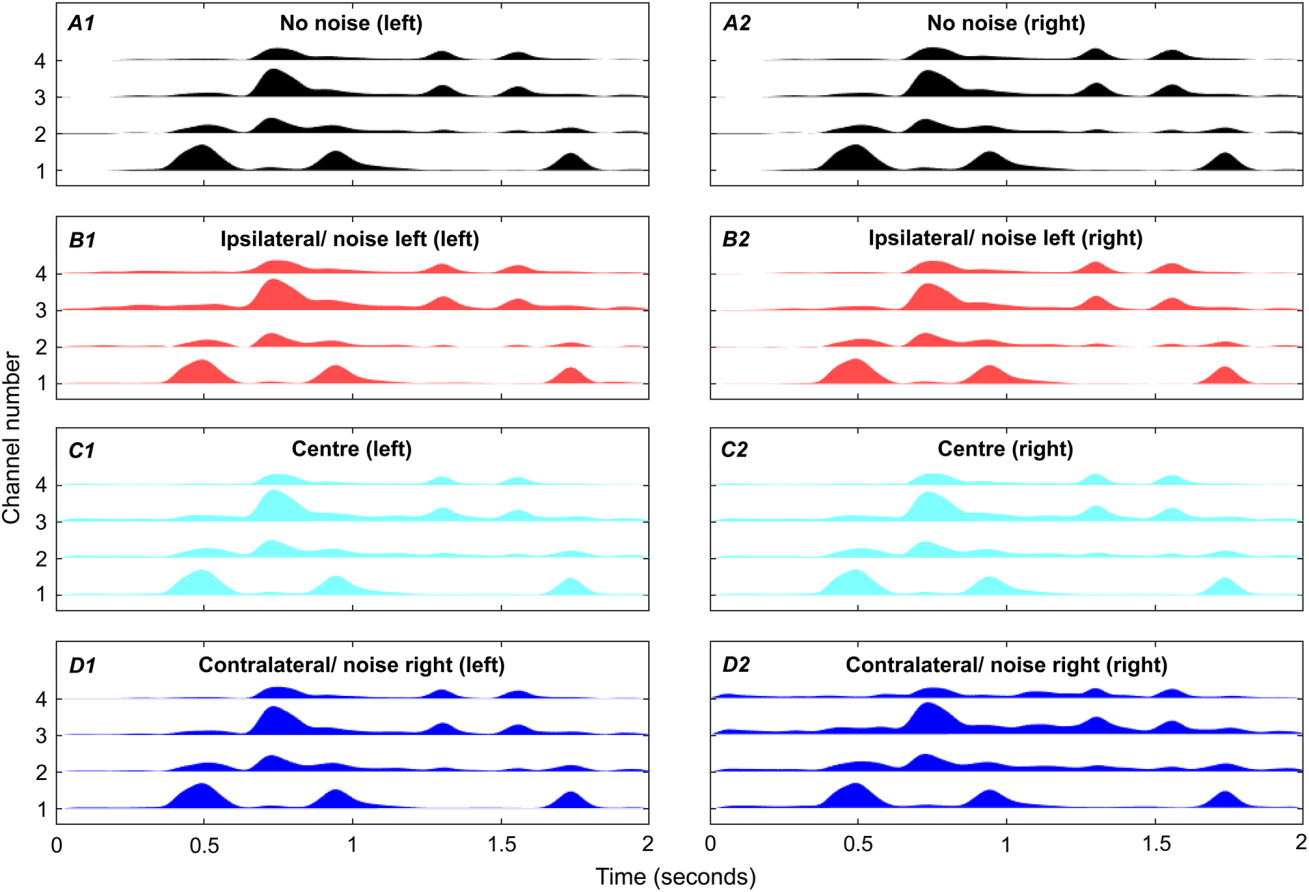
Amplitude envelopes for haptic stimulation for each of the four frequency channels. The height of each channel waveform corresponds to the amplitude of the signal. In all plots, the signal is shown for the IEEE sentence “A large size in stockings is hard to sell” located straight ahead (0°). Panels (**A1**) and (**A2**) show the haptic signal for the clean speech for the left and right wrists, respectively. Panels (**B1**) and (**B2**) show the signal with the multi-talker noise 90° to the left at a signal-to-noise ratio (SNR) of 13.85 dB (matching the average SNR for audio alone in the ipsilateral noise position), panels (**C1**) and (**C2**) show the signal when the noise is co-located with the speech at 9.69 dB SNR (matching the average SNR for audio alone in the central noise position), and panels (**D1**) and (**D2**) show the signal when the noise is 90° to the right at 7.74 dB SNR (matching the SNR for audio alone in the contralateral noise position).

For the haptic signal, head-related transfer functions (HRTFs) were taken from The Oldenburg Hearing Device HRTF Database^49^ and applied to the speech and noise signals for each of the three loudspeaker positions. The three-microphone behind the ear (“BTE_MultiCh”) HRTFs were used, in order to match a typical CI or hearing-aid signal. Each channel of this stereo signal was then passed through four 3^rd^ order Butterworth crossover filters, with crossover frequencies equally spaced on the equivalent rectangular bandwidth (ERB) scale^50^ between 1,000 and 10,000 Hz. This frequency range contains large ILDs^30^ and significant speech energy^29^. To extract the speech amplitude envelope, the Hilbert envelope was calculated for each frequency band and a two-pole IIR filter was applied with a cut-off frequency of 10 Hz. These signals were then used to modulate the amplitude envelopes of four fixed-phase tonal carriers with centre frequencies of between 50 and 250 Hz (equally spaced on the ERB scale). These were then summed for presentation via the vibrometer with a single contact. This approach was adapted from the method used by Fletcher et al.^8^. Examples of haptic signals for each of the experimental conditions are shown in Fig. 3. The haptic signal was presented at an average acceleration magnitude of 2.2 ms^−2^ RMS. The vibrometers were calibrated using a B&K type 4294 calibration exciter.

### Apparatus

Participants were seated in the centre of the Institute of Sound and Vibration Research small anechoic chamber. Three Genelec 8020C PM Bi-Amplified Monitor System loudspeakers were positioned directly in front of the participants and at 90° to the left and right. The loudspeakers were placed 2 m from the centre of the participant’s head when they were in a sitting position, at approximately the height of their ears (1.16 m). An acoustically treated 20″ widescreen monitor was positioned on the floor 1 m in front of the participant for displaying feedback and instructions. Two *HVLab* tactile vibrometers were placed beside the participant’s chair and were used to deliver the vibrotactile signal to the participants’ wrists via a single rigid 10-mm nylon probe with no surround, to maximise the area of skin excitation. All stimuli were controlled using a custom MATLAB script (MATLAB 2019b, The MathWorks Inc, Natrick MA, USA) via an RME Fireface UC audio interface. The haptic signal was processed in realtime using a custom Max patch (Max version 8.0.8).

During testing, the experimenter sat in a separate control room. The participants’ verbal responses were monitored using a sE Electronics 2200A condenser microphone, placed behind the participant's seat. The signal was amplified by the Fireface UC audio interface, and played back through Sennheiser HD 380 Pro headphones. Participants were also monitored visually using a Microsoft HD-3000 webcam.

### Procedure

The experiment was conducted over two sessions, which were not more than 4 days apart (average number of days = 1.8, *SE* = 0.34). Before the training session, the participant first filled out a health questionnaire^9^ and had their vibrotactile thresholds measured. Participants then completed a training session, which lasted around one hour (30 min with EHS and 30 min without). A separate testing session, also lasting around one hour, was completed on a separate day.

In both the training and testing sessions, each participant’s SRT was measured with and without haptic stimulation in three noise conditions: (1) with the speech and noise both played from the loudspeaker directly in front of the participant; (2) with the speech in front and the noise played 90° to the side of the participant’s implant; and (3) with the speech in front and the noise played 90° to the side opposite the participant’s implant. Different speech corpuses were used for training and testing (see Stimuli section). In the training session, trials were marked as correct if participants correctly repeated at least 2 out of 3 keywords, and, in the testing session, trials were marked as correct if participants repeated at least 3 out of 5 keywords. This follows standard practice for testing with each speech corpus. For each SRT in noise, the SNR of the first trial was 20 dB. A one-up, two-down adaptive tracking procedure with a step size of 5 dB was then used for the first two reversals; step sizes of 2.5 dB were used for all subsequent reversals. In the training session, the track was stopped after 25 trials, and in the testing session, the track was stopped after 10 reversals. For the testing session, the SRT was calculated as the mean of the last 8 reversals. In both training and testing sessions, there were two repeats of each condition. For each repeat respectively, conditions were measured in a random order. For both training and testing, participants were given a break of at least 15 min at the halfway point, after all conditions had been completed once.

Responses were made verbally and recorded by the experimenter in the control room. The experimenter was blinded to the location of the noise stimuli. A text display was used to instruct participants to repeat if their response was unclear. In the training session, the sentence text was displayed after the participant response had been recorded. In the testing session, no feedback was provided. In each trial, the participant was instructed to fixate on the central loudspeaker (marked with a red cross), and to keep their head still. For the conditions with haptic stimulation, the message “Please put your wrists on the shaker contacts” was displayed before the trial began; for the conditions without haptic stimulation, the message “Please put your hands on your lap” was displayed. Participants were monitored via a webcam to ensure that they did not move their head, were using the vibrometers in the haptic stimulation conditions, and were not making contact with the vibrometers in the audio-only condition. The vibrometers were near silent, but were active in all conditions to control for any subtle audio cues.

The experimental protocol was approved by the University of Southampton Ethics Committee (ERGO ID: 48820) and the UK National Health Service Research Ethics Service (Integrated Research Application System ID: 265606). All research was performed in accordance with the relevant guidelines and regulations.

### Statistics

The results were analysed using a repeated-measures analysis of variance (ANOVA), with factors ‘Condition’ (with or without EHS) and ‘Noise location’ (ipsilateral, central, or contralateral). Mauchley’s test indicated that the assumption of sphericity had been violated for the interaction between EHS and noise location (*χ*^*2*^(2) = 7.99, *p* = 0.030) so a Greenhouse–Geisser correction was applied (ε = 0.613). The ANOVA used an alpha level of 0.05. Three planned post-hoc two-tailed *t*-tests were conducted (with a Bonferroni-Holm correction for multiple comparisons) to investigate the effect of EHS for each noise location. Three additional post-hoc Pearson’s correlations were also conducted (also corrected for multiple comparisons) to establish whether there was a relationship between EHS benefit and overall SNR.

## Data Availability

The dataset from the current study is publicly available through the University of Southampton’s Research Data Management Repository: 10.5258/SOTON/D1474.
